# A Multi-Modal, Multi-Temporal, Multi-Resolution Benchmark Dataset for Building Height Estimation

**DOI:** 10.1038/s41597-025-06495-3

**Published:** 2025-12-31

**Authors:** Ritu Yadav, Andrea Nascetti, Yifang Ban

**Affiliations:** KTH Royal Institute of Technology, Division of Geoinformatics, Stockholm, Sweden

**Keywords:** Databases, Sustainability

## Abstract

Building heights are crucial for sustainable urban planning and monitoring. While traditional methods use airborne stereo images and LiDAR data for accurate height estimation, their large-scale application is costly and slow, limiting the ability to conduct frequent large-scale monitoring. In contrast, satellite data offers a scalable alternative, further improved with Deep Learning (DL) models. However, the lack of representative open-source training datasets has constrained the progress in this field. In this paper, we introduce M4Heights, a multi-modal, multi-resolution, and multi-temporal dataset designed for building height estimation, spanning diverse architectural styles, urban densities, and terrain complexities across Estonia, Netherlands, and Switzerland. The dataset includes  ≈ 1 million images, comprising time series of Sentinel-1 SAR and Sentinel-2 MSI satellite data, high-resolution aerial orthophotos, and high-quality building height reference maps. Additionally, M4Heights provides the largest associated multi-image super-resolution dataset to enhance height estimation accuracy. Our dataset supports a range of modeling approaches, offers extensibility to new geographic regions and provides opportunities to advance the development of DL models for building height estimation.

## Background & Summary

Building height is a fundamental indicator of urban development, reflecting the extent of residential, commercial, and industrial expansion in a city. Rapid urbanization necessitates the construction of high-rise buildings to accommodate growing populations and economic activities, minimizing land consumption. However, this vertical expansion significantly increases energy consumption. Dense high-rise areas exacerbate urban heat island effect, further increasing energy demand. Buildings account for over 35% of global energy consumption, with urban centers responsible for up to 90% of this usage^1,2^. Overall, buildings are responsible for 21% of global greenhouse gas emissions and 30% of global CO2 emission^3^. Along with building energy consumption and urban heat island effect, accurate building height data is also vital for diverse applications like air pollution modeling, population estimation, digital twin mapping, and telecom network planning^4–6^. However, despite its importance, comprehensive and up-to-date building height information remains scarce, particularly in large-scale applications.

Traditionally, building heights are derived from high-resolution data sources such as airborne Light Detection and Ranging (LiDAR) and optical imagery, which have facilitated numerous height estimation methods^7–10^. However, data collection is expensive and has limited coverage. Recent efforts, such as EUBUCCO^11^, provided a scientific database of 200 million building footprints across 27 European Union countries and Switzerland, but only 8 of them have complete building height coverage. Moreover, while some of these heights are derived from high-resolution data, others come from less reliable or unknown sources, highlighting the lack of comprehensive three-dimensional building information even in data-rich regions like Europe. On a global scale, Microsoft provides building height^12^, leveraging high-resolution satellite imagery in addition to airborne data. However, the dataset is limited to large cities and is not fully open-access^13^. The high associated costs of high-resolution data and limited coverage make these approaches impractical for large-scale applications and their timely updates. Moreover, if building heights can be derived using globally available open-source data, the methods could be adopted by developing countries where collecting high-resolution data is more challenging.

In the past decade, several studies have explored open-source remote sensing data for height estimation at varying spatial resolution (1 km - 10 m) and scale, from city to global level. Building height estimation products such as GHSL-Built-H R2023A^14^ and WSF-3D^15^ provide building heights globally, but their low spatial resolution (90-100 m) limits their utility for detailed applications. Moreover, WSF-3D is based on a commercial high-resolution TanDEM-X product (2011-2013), making it outdated and costly to update. The spatial resolution is improved (30 m to 10 m) with the addition of multi-modal and other auxiliary data^13,16–20^. Furthermore, the precision has improved from evolving models, from traditional machine learning models such as random forest, XGBoost, and SVM^17,19,21^ to DL models such as U-Net variations, transformers and their fusions^22–25^. However, existing datasets and models have some key limitations listed below. We introduce M4Heights^26^, a benchmark dataset designed for large-scale building height estimation at 10 m resolution to address these limitations. Covering  ≈ 6 million hectares across Estonia, the Netherlands, and Switzerland, M4Heights leverages open-source remote sensing data to provide a scalable and cost-effective dataset. We identify three major limitations in existing datasets and outline how M4Heights addresses them:

1. **Lack of benchmark datasets:** while some datasets (e.g., EUBUCCO^11^) provide building height reference derived from traditional methods, and others (GHSL-Built-H R2023A^14^, WSF-3D^15^, Microsoft^12^, Google 2.5D etc.) offer model-predicted heights, there are no DL-ready training datasets for large-scale building height estimation i.e, none of these datasets provide input data and references organized to train deep learning models. To the best of our knowledge, M4Heights is the first publicly available, large-scale, DL-ready benchmark dataset, facilitating training new models, comparisons and expansion to new regions.
2. **Limited utilization of multi-temporal data:** existing studies^14,17,18^ commonly leverage the spatial and spectral dimensions, underutilizing the temporal dimension despite its positive correlation with building height. M4Heights provides 12-month time series of Sentinel-1 and Sentinel-2 images for each data record, encouraging further research in the temporal aspect.
3. **Dependence on high-resolution data:** most existing approaches that use high-resolution data rely on them as inputs, making these approaches costly with limited scalability and adaptability to new regions. Recognizing the importance of fine spatial details in dense urban areas, we provide 1 m aerial orthophoto images for each instance. Instead of using these images as direct inputs, M4Heights encourages their use in super-resolution tasks, allowing models to enhance feature learning across varying levels of granularity without relying on high-resolution imagery in unseen regions.

## Methods

### Data Collection and Pre-processing

**Fig. 1:**
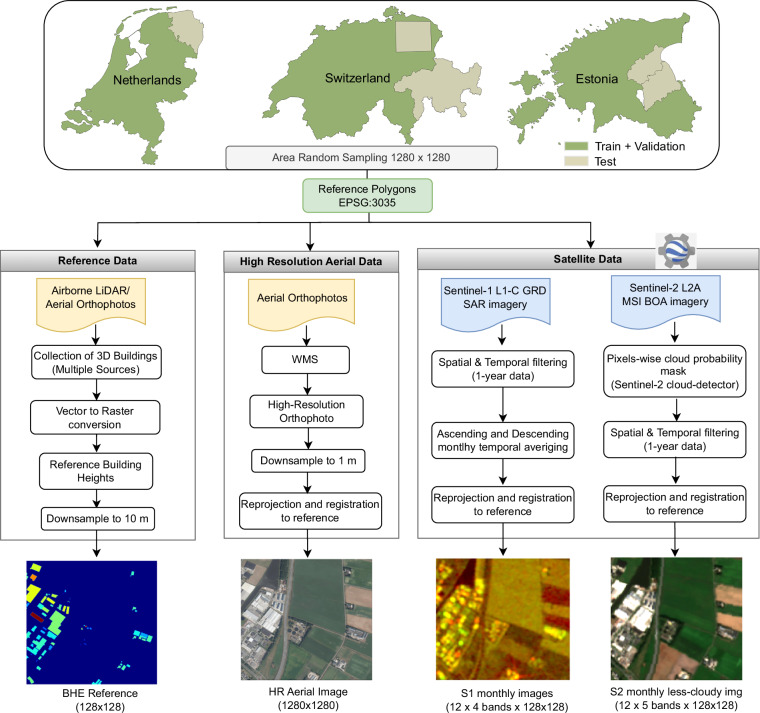
Data Collection Framework.

M4Heights^26^ dataset contains multi-modalities (Sentinel-1 SAR, Sentinel-2 multispectral), multi-resolution (Sentinel-1&2 at 10 m and aerial orthophoto at 1 m spatial resolution), multi-temporal (time series of Sentinel-1&2 data) and supports multi-tasks: building height estimation and multi-image super-resolution. The dataset encompasses buildings of diverse architectural styles, densities, and height distributions across countries with varying terrain complexities. Estonia is characterized by rolling hills, numerous islands, and dense forests. Buildings are relatively sparse and predominantly low-rise. Netherlands, A flat, low-lying country with dense urban areas and generally buildings are taller than in Estonia. Switzerland features a highly complex terrain, including dramatic alpine regions and mountainous landscapes. Urban areas in Switzerland exhibit higher densities and wider ranges of building heights compared to both Estonia and the Netherlands. The technical details of the benchmark dataset are summarized in Table 1 and data collection steps are outlined in Fig. 1. A detailed description of the data collection, filtering and organization is given in the following subsections.

**Table 1:** Dataset Specifications.

	Reference	Modality 1	Modality 2	High Resolution
**Sensor**	ALS/Aerial Orthophoto	SAR	MSI	Aerial Orthophoto
**Source**	ELB, 3DBAG, Swisstopo	Sentinel-1 (ESA)	Sentinel-2 (ESA)	NGR, Swisstopo, ELB
**Source Resolution**	8-18 *p*/*m*^2^, 10-25 cm	10 m	10 m, 20 m	10-25 cm
**Target Resolution**	10 m	10 m	10 m	1 m
**Channels**	height	VV, VH (ASC, DSC)	B2, B3, B4, B8, B12	RGB
**Coverage**	≈6 million hectares covering Estonia, Netherlands, Switzerland			
**Data Size**	≈1 million files

#### Reference Data

The reference building height maps are either based on airborne LiDAR or stereo aerial imagery combined with local cadastral data. For Estonia, the reference building height maps are based on airborne LiDAR. The data mission was conducted by Estonian Land Board (ELB) https://xgis.maaamet.ee/xgis2/page/app/maainfo. The flights were scheduled every summer to cover part of the country. Following a four-year cycle (2017-2020), the entire Estonia was covered by LiDAR with a point density of 18 *p*/*m*^2^. The generated point cloud was automatically classified, interpolated and combined with building footprints from local cadastral data to derive building heights defined as ground to maximum building height. The data is verified and corrected by the land administration.

For the Netherlands, the reference building height maps are also derived from airborne LiDAR with 8 *p*/*m*^2^ point density. The data was acquired between 2014-2019 under a mission by Actueel Hoogtebestand Nederland (AHN) https://www.ahn.nl/. The data is automatically classified and integrated with the 2D building footprints from Buildings and Addresses Register. On top of this, Level Of Details (LOD) models were prepared by 3Dbag, TUDelft https://3dbag.nl/en/viewer. The LOD models were validated and updated using footprint, building registration information from the national registers of buildings to ensure high quality. Our reference height maps are directly fetched from the LOD 1.2 model. The building height is provided with 0th (minimum height), 50th (median), 70th and 100th (maximum) percentile roof inclusion. Previous studies^27^ have proposed using the 70th percentile as a reliable representative of constant building height. It provides a balanced approach, capturing the height value while avoiding outliers, and serves as a reasonable approximation of the overall height of buildings. Therefore, we used the 70th percentile as the reference building height.

The Switzerland building height reference maps are based on stereo aerial imagery captured over entire country by the Swiss Federal Office of topography (Swisstopo) between 2018 and 2021 https://www.swisstopo.admin.ch/en/maps. The aerial imagery has a Ground Sampling Distance (GSD) of 10 cm, providing detailed optical information to capture accurate height from stereo pairs. The aerial digital photo serves as the base data for the extraction and modeling of the buildings. The building heights are extracted using the photogrammetric method of stereo-image correlation. The height of the building is taken as the height from ground to the lowest roof point.

All the data sources in Table 2 provide building height data under **CC BY 4.0** license. Table 2 also provides the reference data collection years (Ref. years) and the sensors used to derive building heights for each country. We have collected building heights from the mentioned sources and to ensure consistency, all reference building height maps have been projected to a common coordinate system, EPSG:3035 (LAEA Europe), which is the European terrestrial reference system.

**Table 2:** Reference Building Height Specifications.

	Ref. years	Sensor	Data Source
**Estonia**	2017-2020	airborne LiDAR	Estonian Land Board
**Netherlands**	2014-2019	airborne LiDAR	Actueel Hoogtebestand Nederland
**Switzerland**	2018-2021	stereo aerial orthophoto	Swiss Federal Office of Topography

#### Satellite Data

The input satellite data contains 12-month time series of Sentinel-1 SAR and Sentinel-2 MSI, both at 10 m spatial resolution. The ESA Sentinel-1 mission captures dual-polarization (HH+HV and VV+VH) C-band SAR data at 5.405 GHz. The captured scenes are pre-processed using the Sentinel-1 toolbox^28^ to generate a calibrated, ortho-corrected GRD product. The pre-processing steps include thermal noise removal, radiometric calibration, and terrain correction followed by log scaling ($10\,\log \,(x)$) to convert the values to decibels. We used all dual-band VV+VH scenes acquired in interferometric wide swath mode over each reference patch area during 12-month period. We downloaded the data captured in both ascending (ASC) and descending (DSC) passes to make use of incidence angle’s influence on the backscatter coefficient, as their fusion can significantly mitigate the impact of inherent geometric distortions in SAR images^29^. Monthly mean images of VV and VH polarizations for both passes were computed using pixel-wise temporal averaging to reduce SAR speckle noise. The output bands VV_ASC, VH_ASC, VV_DSC and VH_DSC, were saved in a single geotiff file.

The ESA Sentinel-2 mission captures optical images with 13 spectral bands at 60 m, 20 m and 10 m spatial resolutions. We collected five bands at 10 m spatial resolution, namely B2 (blue), B3 (green), B4 (red), B8 (near infrared) and B12 (shortwave infrared), as the signals in these bands are more relevant to buildings. Harmonized level-2A bottom-of-atmosphere (BoA) reflectance was chosen over normal BoA Sentinel-2 images to take the benefit of improved radiometric resolution through a new processing baseline. Less cloudy monthly composites are generated using cloud percentage in each patch. Given that 1280 m × 1280 m is a small portion of the Sentinel-2 image footprint, the cloud percentage from the metadata is not a reliable measure for local cloud percentage. Therefore, we used Sentinel2-cloud-detector library to generate pixel-wise cloud probability masks^30^ which are used to calculate the local percentage of clouds for each reference patch. Similar to Sentinel-1, the five bands of Sentinel-2 namely B2, B3, B4, B8 and B12 are saved in one geotiff file. Satellite data was pre-processed and downloaded using Google Earth Engine (GEE)^31^ python API https://github.com/google/earthengine-api.

#### High Resolution Aerial Images

The benchmark dataset is further enriched by adding high-resolution aerial orthophotos corresponding to all reference patches. Nationwide high-resolution imagery is collected every summer in the Netherlands, Estonia, and Switzerland over one to four-year period. Estonia and the Netherlands also conduct additional image collection in the spring months. The data is captured by Leica Geosystems ADS100 camera, which is a strip sensor camera with radiometric resolution of 12 bit and is capable of capturing images up to 3cm resolution. The camera simultaneously records four channels (Red+Green+Blue+NearIR) from three views: nadir, rear and forward. The stages of orthophoto production are aerial imaging flight design, installation of markings, aerial photography (flying) and post-processing of data on the ground which includes calculation of flight trajectories, triangulation, mosaicing and compilation of orthophotos.

The data is captured at different spatial resolutions (8 cm, 10 cm, 25 cm) and spectral bands (RGB, RGB+NIR) depending on the data collection season and country. The processed data is available at the sources mentioned in Table 2 through Web Map Tile Service (WMTS) and Web Map Service (WMS). All the data is available under a **CC BY 4.0** license. For consistency, we selected RGB orthophotos at 25 cm resolution from the summer collection across all three countries. Data was downloaded using Web Map Tile Service (WMTS) in the OWSLib https://github.com/geopython/OWSLib/ library designed to handle geospatial data. The script is developed to efficiently download three-channel (RGB) high-resolution data followed by reprojection to EPSG:3035 and co-registration with the reference patches.

#### Data Sampling, Filtering and Splits

The benchmark dataset was generated following several steps highlighted in the data collection framework reported in Fig. 1. First, training and test areas were defined within individual countries. The test areas were chosen to include a diverse mix of environments, from complex terrain to flat landscapes, dense city centers with high-rise, closely packed buildings as well as rural settlements where buildings are low-rise and sparse. The remaining area is used for training. Both training and test areas are sampled with 1280 m × 1280 m patches using area random sampling. Patches are sampled with an overlap of 20% to ensure comprehensive coverage. The train and test dataset is collected from separate areas to avoid any evaluation biases. The train and test areas are shown in Fig. 1.

Reference height maps were generated for all sampled patches, provided that each patch contained at least five buildings. With this filtering, we avoid numerous patches with rare or no buildings, resulting in a more balanced dataset. Building height maps were recorded as continuous values starting from zero. Since it is improbable to have a building with less than 1.0 m of height, we adjusted any values below 1.0 m to zero.

For each reference building height map, we collected 12-month time series data from Sentinel-1 SAR and Sentinel-2 MSI, as well as high-resolution aerial orthophotos from the final year of the reference data collection period (“Ref. years” Table 2), which is 2019 for Estonia, 2018 for the Netherlands and 2020 for Switzerland. As the urban growth rate of Estonia, the Netherlands, and Switzerland during “Ref. years” period was under 2 percent, we anticipate minimal impact on the quality of the dataset.

In the end, since our Sentinel-1/2 satellite data is at 10 m spatial resolution, the reference building height maps are resampled to 10 m spatial resolution using inter-area interpolation and high-resolution orthophotos are resampled to 1 m spatial resolution. All dataset patches were reprojected to EPSG:3035 using mathematically exact transformations via rasterio, introducing no additional shift. Residual misalignments stem from original dataset co-registration (<6 m for Sentinel-1/2, 10-100 cm for orthophotos)^32^, which remain sub-pixel relative to our 10 m target task resolution and primarily affect building-edge pixels, minimally impacting overall height regression. After processing and filtering, the dataset contains 37,100 data records. The number of train and test records for each country is given in Table 3. The train set was further split into training and validation sets with an 80/20 random split.

**Table 3:** Reference Building Height Specifications.

	# Reference	# S1 (12 × #Reference)	# S2 (12 × #Reference)	# Aerial	Area (hectare)
**Train+Val**	31507	378084	378084	31507	5162107
**Test**	5593	67116	67116	5593	916357
**Total**	37100	445200	445200	37100	6078464

### Building Height Estimation Models

**Fig. 2:**
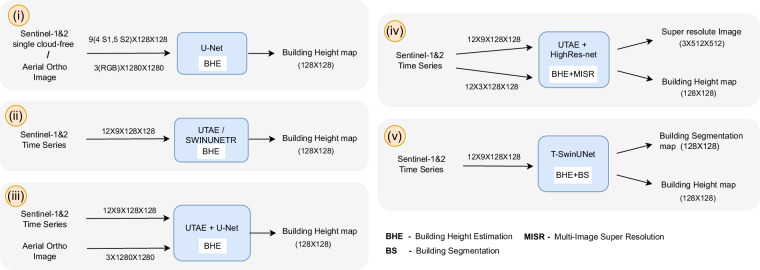
Depiction of model experiments.

The section focuses on designing baselines for the M4Heights^26^ dataset, using a combination of 10 m Sentinel-1&2 and 1 m high-resolution aerial images as input and estimating building height maps at 10 m spatial resolution. We conducted the following experiments with different input data combinations and training tasks, also summarized in Fig. 2.

**Fig. 3:**
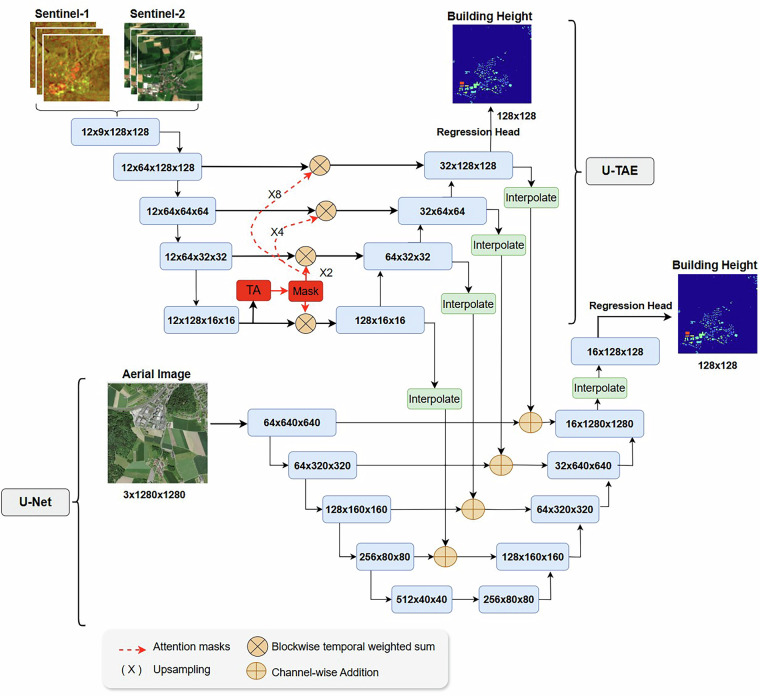
Network architecture of U-TAE+U-Net.

- **(i) BHE on single temporal data:** We trained a U-Net baseline with Resnet34 backbone on Building Height Estimation (BHE) as a regression task. One U-Net model on single Sentinel-1 (S1) and cloud-free summer Sentinel-2 (S2) images with stacked bands four S1 bands (ASC VV, ASC VH, DSC VV, DSC VH) and five S2 bands (B2, B3, B4, B8, B12). Another U-Net model is trained on a High-Resolution aerial ortho image (HR) with three RGB channels. Both models predict building height maps at 10 m spatial resolution (size 128  × 128).
- **(ii) BHE on (S1,S2) TS:** In this experiment, we establish baseline on Sentinel-1&2 time series (TS) data. Two recent DL models SwinUNETR^33^ and UTAE^34^ are trained on 12-month time-series of Sentinel-1&2 for building height estimation as a regression task. SwinUNETR is the SWIN transformer-based U-Net model for segmenting 3D input data. The model replaces U-Net encoder with a Swin transformer, adding the capability to capture long-range dependencies and global context of the image in the learning process. U-TAE is a U-Net specifically designed to handle satellite time-series data. The model extracts rich and adaptive multi-scale spatiotemporal features through a shared encoder and Lightweight Temporal Attention (LTAE^35^). The extracted features are then processed through a spatial decoder. Both SwinUNETR and UTAE have previously demonstrated top performance in benchmarking Sentinel-1&2 time series datasets to learn vertical dimension features^36^ and the official implementations of the models are open-sourced. We used the standard configuration of these models. The segmentation head of the models is replaced with a regression head. The input of the model consists of 12 time-series images of size 128 × 128 pixels and each image contains nine bands: four S1 bands and five S2 bands. The output is a building height map of size 128 × 128 pixels.
- **(iii) BHE on (S1,S2) TS + HR:** In this experiment, we investigate whether the 10 m Sentinel-1&2 time series can improve the estimations from a single high-resolution aerial ortho image. To uncover this, two models, U-TAE and U-Net are trained to estimate building height. The two models are integrated by fusing their decoders as shown in the network architecture (Fig. 3). The decoder stage outputs of UTAE are interpolated (bicubic) and added to the input of the U-Net decoder. The U-TAE branch of the model takes time series of Sentinel-1&2 images (size 12 × 9  × 128  × 128) and U-Net branch takes high-resolution aerial images (3 × 1280  × 1280) as input. Both branch output height maps of size 128  × 128.
- **(iv) BHE+MISR on (S1,S2) TS:** High-resolution optical images, whether aerial or satellite, offer rich spectral, spatial, and structural information, making them widely used for building height estimation^37,38^. Even with a single high-resolution optical image, it is possible to retrieve accurate building height^22^. However, unlike Sentinel-1&2, high-resolution images are often unavailable or very expensive. To deal with such scenario, we utilize an additional task of Multi-Image Super-Resolution (MISR) in which the model learns to construct high-resolution images from multiple low-resolution images, thereby eliminating the need of high-resolution images at inference time. In this experiment, the model performs two tasks: BHE and MISR on input Sentinel-1&2 time series. UTAE^34^ and HighRes-net^39^ models are integrated for the task. HighRes-net is a multi-image super-resolution model, that applies 4  × super-resolution on a sequence of satellite images. HighRes-net uses residual channel attention to model the temporal relationships between low-resolution images. We used a recently proposed version of this model^40^ where the residual channel attention is replaced by LTAE, resulting in better super-resolution. The two models are integrated using simple decoder fusion (Similar to Fig. 3). The decoder stage outputs of the HighRes-net are interpolated (bicubic) and added to the input of the UTAE decoder. The model is trained end to end with time series input along with building height map and high-resolution (2.5 m) aerial images as targets. U-TAE branch of the fusion model takes Sentinel-1&2 time series as input (12 × 9  × 128 × 128) and predicts a single building height map of size 128 × 128. HighRes-net branch takes an input of size 12 × 3 × 128 × 128, where the three channels correspond to B4, B3 and B2 bands of Sentinel-2. Then, use 4 × super-resolution to predict an enhanced image at 2.5 m spatial resolution with size 3 × 512 × 512.
- **(v) BHE+BS on (S1,S2) TS:** In this experiment, the T-SwinUNet model is trained on Sentinel-1&2 time series input and instead of MISR, Building Segmentation (BS) is learned as a parallel task. T-SwinUNet model incorporates a shared CNN and Swin Transformer encoder, temporal attention mechanisms, and a novel feature decoder, all designed to efficiently extract spatiotemporal features from time series data. The model takes Sentinel-1&2 time series (size 12 × 9  × 128  × 128) as input and predicts building height and segmentation map of size 128  × 128 pixels.

#### Evaluation Method

The building height predictions are evaluated using three metrics: Root Mean Square Error (RMSE), *R*^2^ and Intersection over Union (IoU). RMSE indicates the accuracy of predicted heights with respect to the reference and *R*^2^ measures the effectiveness of the model to capture the variance in predictions. The alignment of predicted buildings with reference segmentation map is evaluated IoU metric. The reference segmentation is calculated by binarizing building height maps with a threshold of 1.0 meters, as it is rare to have buildings shorter than 1.0 m.

All metrics are calculated on only building pixels. A good building height estimation model should show low RMSE, high *R*^2^ and high IoU. The metrics RMSE, *R*^2^ and IoU are given in Equations (1)– (3), where *BH*_*ref*,*i*_ represents reference building height, *BH*_*pred*,*i*_ represents predicted building height, TP is true positives, FP is false positives, and FN represents false negatives.

1 $$RMSE=\sqrt{\frac{{\sum }_{i=1}^{n}{(B{H}_{ref,i}-B{H}_{pred,i})}^{2}}{n}}$$

2 $${R}^{2}=1-\frac{(n-1){\sum }_{i=1}^{n}{(B{H}_{ref,i}-B{H}_{pred,i})}^{2}}{(n-2){\sum }_{i=1}^{n}{(B{H}_{ref,i}-B{H}_{pred,i})}^{2}}$$

3 $${\rm{IoU}}=\frac{{\rm{TP}}}{{\rm{TP}}+{\rm{FP}}+{\rm{FN}}}$$

## Data Records

**Fig. 4:**
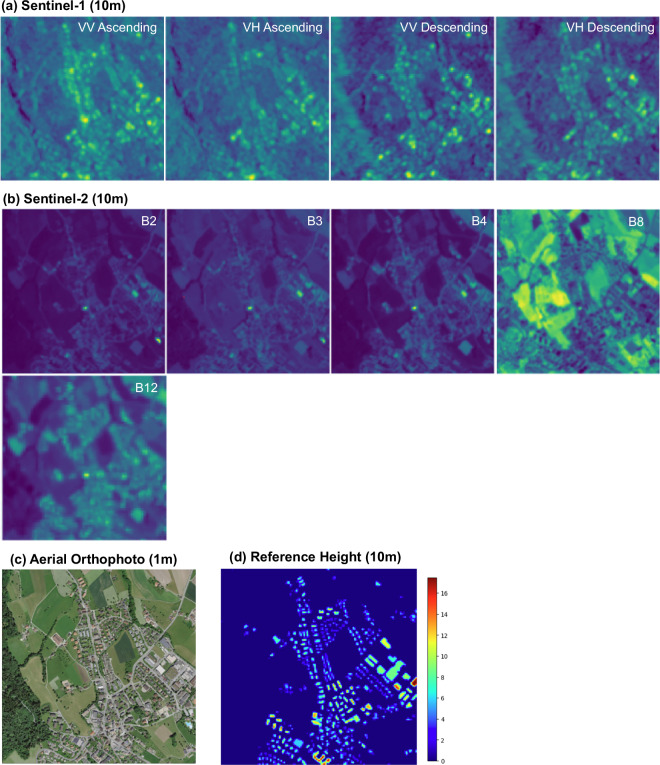
Data Record: (**a**) Sentinel-1 image bands VV_ASC, VH_ASC, VV_DSC, VH_DSC are visualized (**b**) Sentinel-2 bands B2, B3, B4, B8, B12, (**c**) aerial orthophoto as RGB and (**d,** **e**) reference height maps are visualized at (d) 10 m spatial resolution using color gradient. The height values are continuous values starting from 0. The color gradient represents building height in meters.

**Fig. 5:**
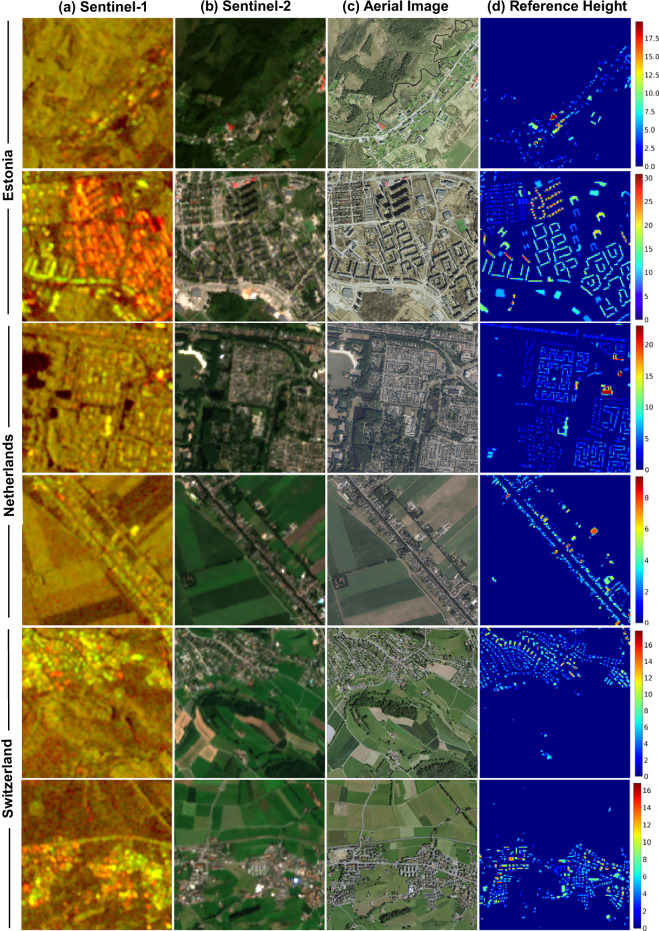
More Data Records: The (**a**) Sentinel-1 images are visualized as (VV asc, VH asc), (**b**) Sentinel-2 as (B4, B3, B2), (**c**) aerial orthophoto as (RGB) and (**d**) reference height as continuous values starting from 0. The color gradient represents building height in meters.

M4Heights dataset is available at the Hugging Face repository^26^. The dataset contains a total of 37100 records, each record represents a ground area of 1280*m* × 1280*m* and is composed of 12 Sentinel-1 time series images at 10 m spatial resolution, 12 Sentinel-2 time series images at 10 m spatial resolution, one aerial orthophoto at 1 m spatial resolution and one reference building height map at 10 m spatial resolution. A sample data record is visualized in Fig. 4. For simplification, we only visualize one cloud-free July month Sentinel-2 and corresponding Sentinel-1 image instead of 12 time series images. Additionally, although Fig. 4 shows only five relevant bands of Sentinel-2 (“B2”, “B3”, “B4”, “B8”, “B12”), we provide 11 bands (“B2”, “B3”, “B4”, “B5”, “B6”, “B7”, "B8”, “B8A”, “B11”, “B12”, ‘probability’) for further exploration. More data samples are presented in Fig. 5, where the visualized Sentinel-1 and Sentinel-2 images are also from July month due to the least cloud presence.

**Fig. 6:**
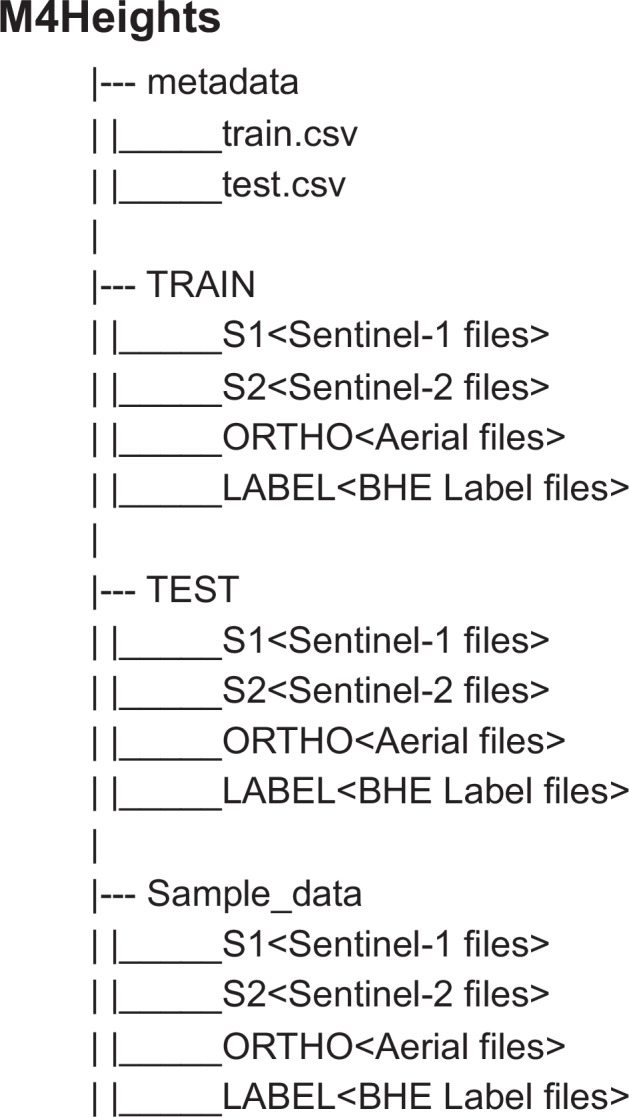
Data Directory Structure.

The dataset contains approximately  ≈ 1 million files, with a total dataset size of around  ≈ 900 GB. The directory structure of the dataset is shown in Fig. 6 and the files are named using the following naming conventions:

- **Label files** :‘img_{country_code}_{Year}_{patch_id}.tif’
- **Aerial files** : ‘img_{country_code}_{Year}_{patch_id}.tif’
- **Sentinel-1&2 files** : ‘img_{country_code}_{Year}_{patch_id}_{month}.tif’

## Technical Validation

This section presents the technical validation of M4Heights^26^ to justify it’s reliability. We first start with the quality of the building height reference map, followed by evaluation of several baseline models trained under different experiment settings addressing the use of dataset in different scenarios. The results from the trained models are discussed in detail to support the technical validity of the dataset.

### Reference Data Validation

The reference building height maps are the official maps provided by the respective national land administrations. These maps are derived from high-resolution aerial images and LiDAR point clouds. The high elevational and positional accuracy of under ±50 cm ensures the precision of reference maps. Additionally, their reliability is further reinforced through manual validation against national building registers conducted by domain experts. The accuracy of the building height references for each country is provided in Table 4.

**Table 4:** Reference Building Height map accuracy.

	Elevation accuracy	Positional accuracy
**Estonia**	±7 cm	±40 cm
**Netherlands**	±15 cm	±30 cm
**Switzerland**	±50 cm	±30 cm

However, building height definitions vary across the three data sources due to how roof height is incorporated, i.e., in Estonia data roof height is included in the building height, in Switzerland roof height is not included and in Netherlands building height is defined by percentiles. Standardizing building height definition would ideally require roof type and precise roof height information^11^, which unfortunately, is not available. This inconsistency in definitions may introduce potential errors in model predictions. However, since the training dataset includes data from all three countries, the model is expected to learn an average representation of roof height, thereby compensating for these differences to some extent. Broadly categorizing building roof types into flat roofs, gable roofs and complex roof structures of historic buildings, flat roofs are unaffected by the definition differences. Gable roofs add approximately one floor (3m) height to the building. Given the three building height definitions with maximum, minimum and 70 percentile roof inclusion, the discrepancy in building height should be between 0 to 3m. For historical buildings with complex roof structures, the discrepancy can be higher; however, such buildings constitute a small fraction of the dataset. The majority of buildings in the dataset have flat, Gable, or similar roof types. Therefore on an average, the induced error is expected between 0 to 1.5m (half a floor). Please note that this error is approximated on the individual building or instance level. At our target resolution of 10 meters, this per-building error is further reduced due to pixel aggregation effects.

### Model Evaluation

We have conducted several experiments to showcase the usage of M4Heights^26^ dataset with several models and training methods. We evaluated models with different input datasets based on modality, resolutions, and temporal dimensions along with models harnessing these inputs. We also experimented with both single and multi-task learning approaches, such as segmentation and super-resolution. The results from our evaluations are presented in Table 5.

**Table 5:** Results from the U-Net and transformer baseline models on the test set under different combinations of inputs.

	Model	Inputs	Reference	Task	RMSE (m)	*R*^2^	IoU
(i)	U-Net^41^	(S1,S2) Single	BH	BHE	4.50 ± 0.025	0.29 ± 0.007	0.46 ± 0.006
	U-Net^41^	HR	BH	BHE	1.54 ± 0.028	0.59 ± 0.010	0.69 ± 0.003
(ii)	U-TAE^34^	(S1,S2) TS	BH	BHE	2.12 ± 0.003	0.46 ± 0.007	0.54 ± 0.006
	SwinUNETR^33^	(S1,S2) TS	BH	BHE	2.13 ± 0.006	0.41 ± 0.005	0.52 ± 0.011
(iii)	U-TAE^34^+U-Net^41^	(S1,S2) TS +HR	BH	BHE	**1.26** ± 0.007	**0.65** ± 0.005	**0.70** ± 0.006
(iv)	U-TAE^34^+HighRes-net^40^	(S1,S2) TS	BH, HR	BHE,MISR	**1.94** ± 0.003	**0.50** ± 0.009	**0.55** ± 0.002
(v)	T-SwinUNet^25^	(S1,S2) TS	BH	BHE,BS	**1.91** ± 0.009	**0.53** ± 0.013	**0.58** ± 0.010

The models are trained and tested on all three sites Netherlands (NLD), Switzerland (CHE), and Estonia (EST). The overall best results are highlighted in bold.

**Fig. 7:**
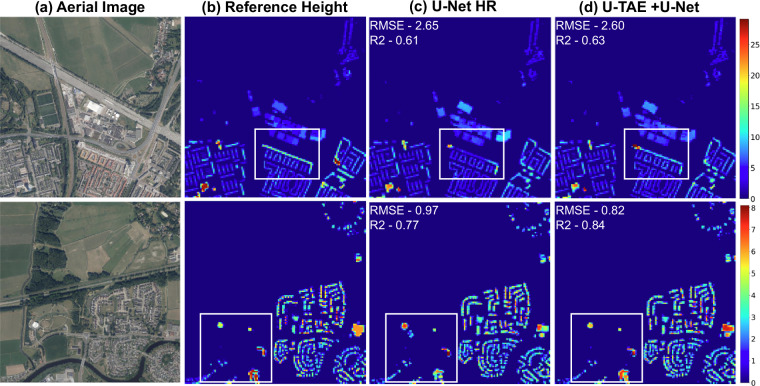
Comparison of (**c**) BHE by U-Net on single temporal HR image and (**d**) BHE by U-TAE+U-Net on (S1,S2) TS + HR images. The color gradient scale represents building height in meters. The major differences areas are highlighted in squares.

The results demonstrate that building height is best estimated using high-resolution images (i), achieving an RMSE of 1.54 and an *R*^2^ of 0.59. This is because a single high-resolution image provides superior spatial and structural details, enabling the model to estimate building height more accurately. Incorporating Sentinel-1&2 time-series (iii) images further improves height estimations, reducing the RMSE to 1.26 and increasing *R*^2^ to 0.65. Although Sentinel images are lower resolution, they provide additional spatio-temporal features from time series data, particularly in optical time series, where gradual changes in building shadows over time provide valuable elevation information^19,21^. For Sentinel-1 SAR, building height affects the magnitude of layover and double-bounce scattering from walls and ground surfaces, and these effects are modulated by changes in look direction and surface wetness across the annual cycle. Compared to using a single image, multiple acquisitions of the same area offer a more detailed vertical profile of buildings^20,37^. These multiple observations increase the amount of per-pixel information, which is harnessed by temporal attentive DL model (U-TAE) to retrieve more accurate features and improve performance on downstream tasks of height estimation. Additionally, the overlap of predicted building height with segmentation achieves a value of 0.70. The comparison of the two experiments is further strengthened by the test samples visualized in Fig. 7.

However, it is crucial to note that high-resolution images are prohibitively expensive and infrequently updated. For example, even in wealthier regions like Europe, such datasets are updated only once every several years. This challenge is even more pronounced in developing or underdeveloped countries, where financial and organizational constraints limit access to high-resolution imagery. In such scenarios, and for large-scale applications, frequently available public datasets such as Sentinel-1&2 offer a viable alternative despite their lower accuracy in height estimation compared to high-resolution images.

**Fig. 8:**
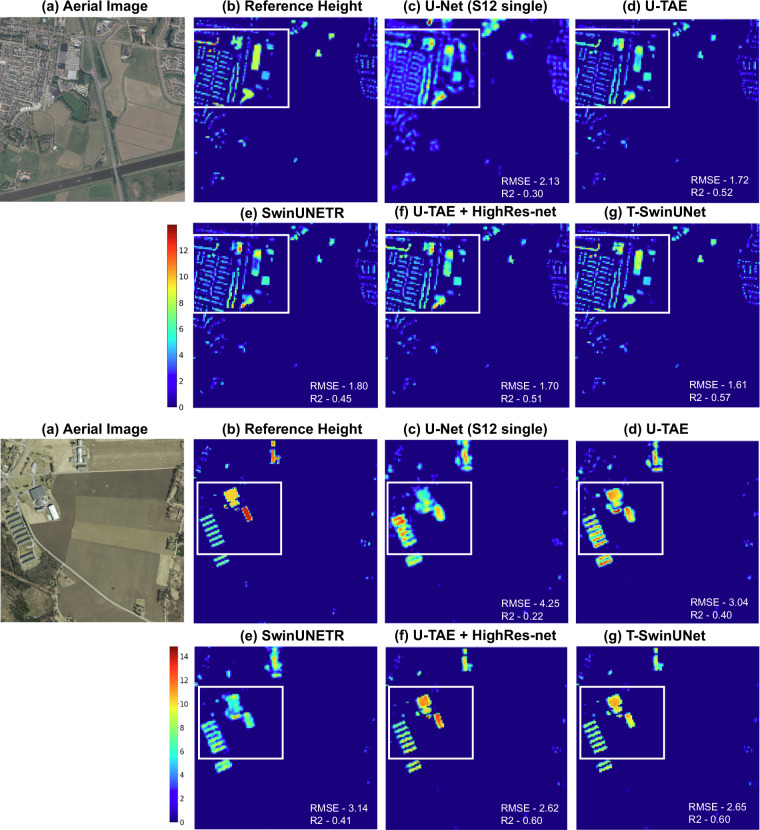
Results samples to compare building height estimations from models trained on Sentinel-1&2 inputs. The color gradient scale represents building height in meters. The major differences are highlighted in squares.

When using only Sentinel-1&2 as input data, a sharp performance improvement is observed with time series input (RMSE 2.12, *R*^2^ 0.46) instead of single Sentinel-1&2 images (RMSE 4.50, *R*^2^ 0.29). Incorporating the super-resolution task further enhances the results (RMSE 1.94, *R*^2^ 0.50), enabling the model to learn building height while capturing detailed high-resolution features through multi-image super-resolution task. Moreover, advancements in network design, such as T-SwinUNet, contribute to better estimation of building heights with 1.91 m RMSE and 0.53 *R*^2^. Result samples are visualized in Fig. 8 for qualitative comparison.

**Fig. 9:**
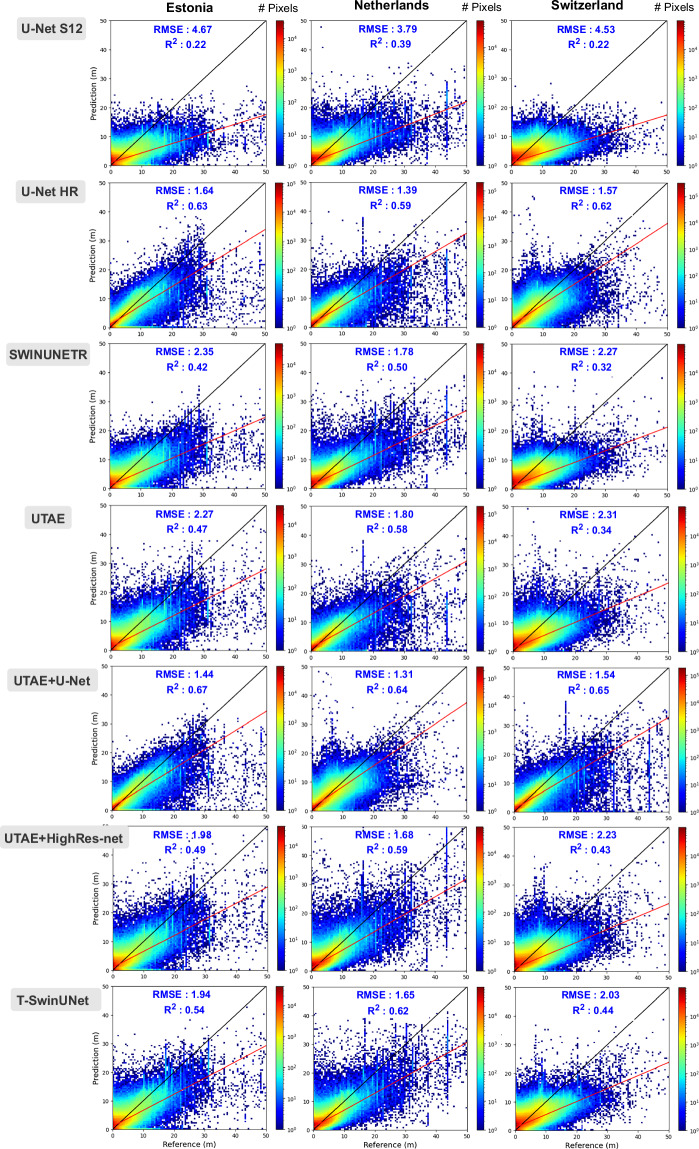
Correlation between predicted building and reference building height at 10 m. The black diagonal plot y = x represents the best possible fit and the red line is the actual fit to the plot.

More in-depth analysis on country-wise results is facilitated through scatter plots (Fig. 9) depicting the correlation between predicted and reference building heights. Overall, the predictions for the low-rise buildings have better correlation with the reference heights and the correlation drops gradually with the height of the buildings. The problem is less severe for models with high-resolution aerial inputs, as they provide access to fine-scale features of building geometries. For the models operating on only Sentinel-1&2 inputs, the best results are achieved on the Netherlands site and the worst on Switzerland. The lower performance in Switzerland is attributed to the country’s high terrain complexity. Adding a good resolution DEM might help alleviate terrain-induced errors and improve model accuracy.

While the dataset can be used with our provided train-test splits, it can also be explored with different train and test data combinations, such as training on any two countries and testing on the third one. Such experiments help to evaluate the generalizability of a trained model to the unseen terrain of a different country. This in turn also identifies the significance of training on regional data samples. Table 6 provides an example of such use case where we trained a couple of models on estimating building heights in Switzerland and Netherlands and tested on Estonia buildings.

**Table 6:** Demonstration of partial data usage.

Model	Inputs	Task	Eval	RMSE (m)	R2	mIoU
SwinUNETR^33^	(S1,S2) TS	BHE	EST	2.68 ± 0.006	0.32 ± 0.001	0.37 ± 0.003
U-TAE^34^	(S1,S2) TS	BHE	EST	2.54 ± 0.022	0.33 ± 0.002	0.42 ± 0.005
U-TAE^34^+HighRes-net^40^	(S1,S2) TS	BHE+MISR	EST	**2.16** ± 0.009	**0.42** ± 0.005	**0.50** ± 0.001

Models are trained on two sites Netherlands, Switzerland, and tested on Estonia (EST) test data.

## Usage Notes

The dataset is provided with train and test splits. The validation set can be customized from the training dataset as per the requirements. All the data point filenames are provided in CSV files under metadata. We have provided several ways to utilize the dataset using several models under different input settings. The code and the instructions are provided on our GitHub repository.

M4Heights^26^ is a dataset designed for building height estimation at 10 m spatial resolution. However, it can also be used for building segmentation at 10 m spatial resolution. In addition, we provide the largest associated dataset for multi-image super-resolution task from Sentinel-2 and Sentinel-2 time series data, indicating that the dataset can also be used for general remote sensing super-resolution tasks.

One of the major advantages of the M4Heights dataset is that it is based on the Sentinel-1 and Sentinel-2 time series which is available globally and frequently. Therefore, M4Heights can be easily expanded to other sites where the building height references are available.

## Data Availability

The proposed M4Heights data set is available at our Hugging Face repository: 10.57967/hf/4765. The dataset contains a total of 37100 records, each record represents a ground area of 1280 m × 1280 m and is composed of 12 Sentinel-1 time series images at 10 m spatial resolution, 12 Sentinel-2 time series images (under S1, S2 folders) at 10 m spatial resolution, one aerial orthophoto (under ORTHO folder) at 1 m spatial resolution and one reference building height map (under LABEL folder) at 10 m spatial resolution. The naming conventions of the files are : **Label files** :‘img_{country_code}_{Year}_{patch_id}.tif’, **Aerial files** : ‘img_{country_code}_{Year}_{patch_id}.tif’, **Sentinel-1&2 files** : ‘img_{country_code}_{Year}_{patch_id}_{month}.tif’. The dataset is divided into training and test set, the data is given in corresponding folders. We also provide sample data (Under Sample_Data folder) for testing.

## Code availability

The codebase to validate the dataset through DL models is available on our GitHub repository: https://github.com/RituYadav92/M4Heights.
